# Elevated plasma Ninjurin-1 levels in atrial fibrillation is associated with atrial remodeling and thromboembolic risk

**DOI:** 10.1186/s12872-022-02593-x

**Published:** 2022-04-07

**Authors:** Chen Fang, Kaicheng Jiao, Kun Zuo, Xinchun Yang

**Affiliations:** Heart Center & Beijing Key Laboratory of Hypertension, Beijing Chaoyang Hospital, Capital Medical University, 8th Gongtinanlu Rd, Chaoyang District, Beijing, 100020 China

**Keywords:** Nerve injury-induced protein 1, Atrial fibrillation, Left atrial volume index, Thromboembolic risk

## Abstract

**Background:**

Nerve injury-induced protein 1 (Ninj1) is elevated in various inflammatory diseases. The soluble form of Ninj1 yield by matrix metalloproteinase cleavage is a secreted protein and inhibits cell adhesion and inflammation. However, the role of plasma Ninj1 in atrial fibrillation (AF) has not been reported. The present study aimed to investigate the correlation between plasma Ninj1 levels and AF.

**Methods:**

A total of 96 AF patients [age 66.00 (60.00, 72.00) years, male 56 (58.33%)] and 51 controls without AF [age 65.00 (55.00, 68.00) years, male 21 (41.18%)] were enrolled in this study. Plasma Ninj1 concentrations were detected using enzyme-linked immunosorbent assay. Also, the clinical characteristics, left atrial volume index (LAVI), CHA2DS2-VASc score, and HAS-BLED score were evaluated.

**Results:**

Plasma Ninj1 levels were significantly higher in patients with AF than in controls (*P* < 0.001). Plasma Ninj1 levels were positively correlated with LAVI (*P* = 0.019) and CHA2DS2-VASc score (*P* = 0.024). Logistic regression analysis confirmed that the Ninj1 plasma levels were associated with AF (*P* = 0.009). The receiver operating characteristic analysis showed that plasma Ninj1 had a predictive value for AF (*P* < 0.001).

**Conclusions:**

Plasma Ninj1 levels were elevated in patients with AF, associated with left atrial enlargement and thromboembolic risk in AF.

**Supplementary Information:**

The online version contains supplementary material available at 10.1186/s12872-022-02593-x.

## Background

Atrial fibrillation (AF) is the most common clinical arrhythmia that contributes to significant morbidity and mortality, incurring a significant societal burden [1]. The occurrence of AF is associated with an increased risk of stroke and congestive heart failure [2]. Several studies indicated that the underlying pathophysiological mechanisms of AF are complex and variable, including atrial fibrosis and inflammation [3].

Nerve injury-induced protein 1 (Ninjurin1, Ninj1) is an adhesion molecule involved in the pathogenesis of inflammatory disease and pulmonary fibrosis [4, 5]. Ninj1 consists of two transmembrane domains, an intracellular region and extracellular N- and C- termini [6]. The N-terminal ectodomain of Ninj1 is liberated by matrix metalloproteinase 9 (MMP9) to yield a soluble form (sNinj) with chemokine-like activity into circulation [6]. Different from Ninj1 located on the cell surface, sNinj1 inhibits cell adhesion in a non-autonomous manner [7]. A recent study reported that sNinj1 is a secreted protein that ameliorates atherosclerosis by regulating inflammation [8]. However, the correlation between circulating Ninj1 levels and AF has not been investigated.

In the current study, we evaluated Ninj1 plasma levels in patients with or without AF, investigated the underlying value of plasma Ninj1 as a biomarker in AF, and explored the association between plasma Ninj1 and thromboembolic risk.

## Methods

### Study cohort

A total of 96 nonvalvular AF patients and 51 individuals without AF were recruited in Beijing Chaoyang Hospital between September 2020 and August 2021. The diagnosis of AF was based on the 2020 guidelines established by the European Society of Cardiology (ESC). The exclusion criteria included patients with congenital heart disease, structural heart disease, acute coronary syndrome, acute or chronic infection, autoimmune disease, renal failure (estimated glomerular filtration rate < 15 ml/min/1.73 m^2^), severe liver dysfunction (a two- to three-times elevation of transaminases) and malignant tumors. The research protocol was approved by the ethics committee of Beijing Chaoyang Hospital. The study conformed to principles of the Declaration of Helsinki and all participants provided informed consent.

### Clinical characteristics

Clinic characteristics, medical history and vital signs of all participants were obtained at enrollment. Electrocardiography, echocardiography, serum indexes of liver and kidney functions, serum lipids, hemoglobin A1C and troponin I were recorded. LAVI was defined as left atrial volume indexed for body surface area. CHA2DS2-VASc and HAS-BLED scores were calculated for thromboembolic and bleeding risk assessment respectively [1].

### Plasma Ninj1 measurement

Peripheral venous blood was drawn from all subjects in the morning and stored in EDTA anticoagulation vacuum tubes. Plasma samples were immediately obtained by centrifugation at 3000 rpm for 10 min, 4 °C and kept at -80℃ until measurement.

The plasma Ninj1 concentration was measured using an enzyme-linked immunosorbent assay kit (CSB-EL015808HU, CUSABIO) following the protocol. The kit used a double-antibody sandwich enzyme-linked immunosorbent assay to determine the level of plasma Ninj1 in the samples.

### Statistical analysis

Continuous variables were presented as the mean ± SD (normal distribution) or median (quartile). Categorical variables were presented as numbers and percentages. Continuous data distributed normally were analyzed using Student's *t*-test and nonnormally distributed data were compared using the Mann–Whitney test. Categorical data were analyzed using the Chi-square test. Pearson and Spearman tests were performed for the correlation between Ninj1 levels and the variables. The receiver operating characteristic (ROC) curves were used to assess the predictive performance of plasma Ninj1. The clinical characteristic associated with AF were analyzed using binary logistic regression. Further, variables with *P* < 0.1 in the single-factor analysis were included in the stepwise multivariable analysis. All statistical analyses were performed with the MedCalc (V19.6.4) and SPSS version 25.0 (IBM Corporation, Armonk, NY). A *P *value < 0.05 (two-sided) were considered statistically significant.

## Results

### Baseline characteristics of subjects

A total of 96 AF patients, including 54 with paroxysmal AF and 42 with persistent AF, and 51 individuals as controls were enrolled in this study. The clinical characteristics of all participants are summarized in Table 1. Age, hemoglobin levels, and left atrial volume index (LAVI) were higher, while left ventricular ejection fraction (LVEF) was lower in AF patients compared to controls (*P* < 0.05). No significant difference was detected in the male gender, body mass index (BMI), hypertension (HTN), diabetes mellitus (DM), coronary artery disease (CAD), creatinine, total cholesterol (TC), or other clinical characteristics between AF patients and controls.

**Table 1:** Baseline clinical characteristics of the study participants with or without atrial fibrillation

	Control	AF	*P* value
Number	51	96	–
AF history, years	-	4.35 ± 6.51	–
Male (%)	21 (41.18%)	56 (58.33%)	0.057
HTN (%)	26 (50.98%)	58 (60.42%)	0.297
DM (%)	7 (13.73%)	26 (27.08%)	0.096
CAD (%)	2 (3.92%)	10 (10.42%)	0.218
Smoking (%)	15 (29.41%)	17 (17.71%)	0.141
Drinking (%)	12 (23.53%)	16 (16.67%)	0.378
Age, years	65.00 (55.00, 68.00)	66.00 (60.00, 72.00)	0.039*
BMI, kg/m^2^	26.23 ± 3.32	25.68 ± 3.27	0.411
WBC, × 10^9^/L	6.31 ± 1.19	6.05 ± 1.43	0.417
HGB, g/L	129.65 ± 14.69	138.77 ± 16.30	0.016*
PLT, × 10^9^/L	221.78 ± 43.40	201.92 ± 60.16	0.139
TC, mmol/L	4.05 ± 0.95	4.05 ± 0.89	0.986
TG, mmol/L	1.08 (0.88, 1.81)	1.18 (0.88, 1.49)	0.873
LDL-C, mmol/L	2.52 ± 0.85	2.62 ± 0.87	0.519
HDL-C, mmol/L	1.19 ± 0.29	1.09 ± 0.32	0.079
AST, U/L	18.55 ± 5.78	20.43 ± 7.49	0.159
ALT, U/L	16.00 (12.25, 20.95)	17.00 (13.25, 23.00)	0.544
sCr, μmol/L	67.48 ± 14.55	70.97 ± 15.01	0.215
cTNI, ng/mL	0.00 (0.00, 0.01)	0.00 (0.00, 0.01)	0.662
HbA1c (%)	5.90 (5.70, 6.20)	5.90 (5.70, 6.55)	0.921
LVEF (%)	68.38 ± 5.20	64.28 ± 6.67	0.001**
LAVI, ml/m^2^	19.16 (15.08, 21.83)	26.90 (19.83, 32.27)	< 0.001***
ESR, mm/h	4.00 (2.50, 9.50)	4.00 (2.00, 8.00)	0.460

Data are presented as mean ± SD, median (quartile) or number (%). ALT, alanine aminotransfease; AST, aspartate aminotransferase; BMI, body mass index; cTNI, cardiac troponin I; CAD, coronary artery disease; DM, diabetes mellitus; ESR, erythrocyte sedimentation rate; HTN, hypertension; HDL-C, high density lipoprotein cholesterol; HGB, hemoglobin; HbA1c, hemoglobin A1c; LDL-C, low density lipoprotein cholesterol; LVEF, left ventricular ejection fraction; LAVI, left atrial volume index; PLT, platelet; sCr, serum creatinine; TC, total cholesterol; TG, triglyceride; WBC, white blood cell

*P < 0.05; **P < 0.01; ***P < 0.001

### Elevated plasma Ninj1 levels in AF patients

**Fig. 1:**
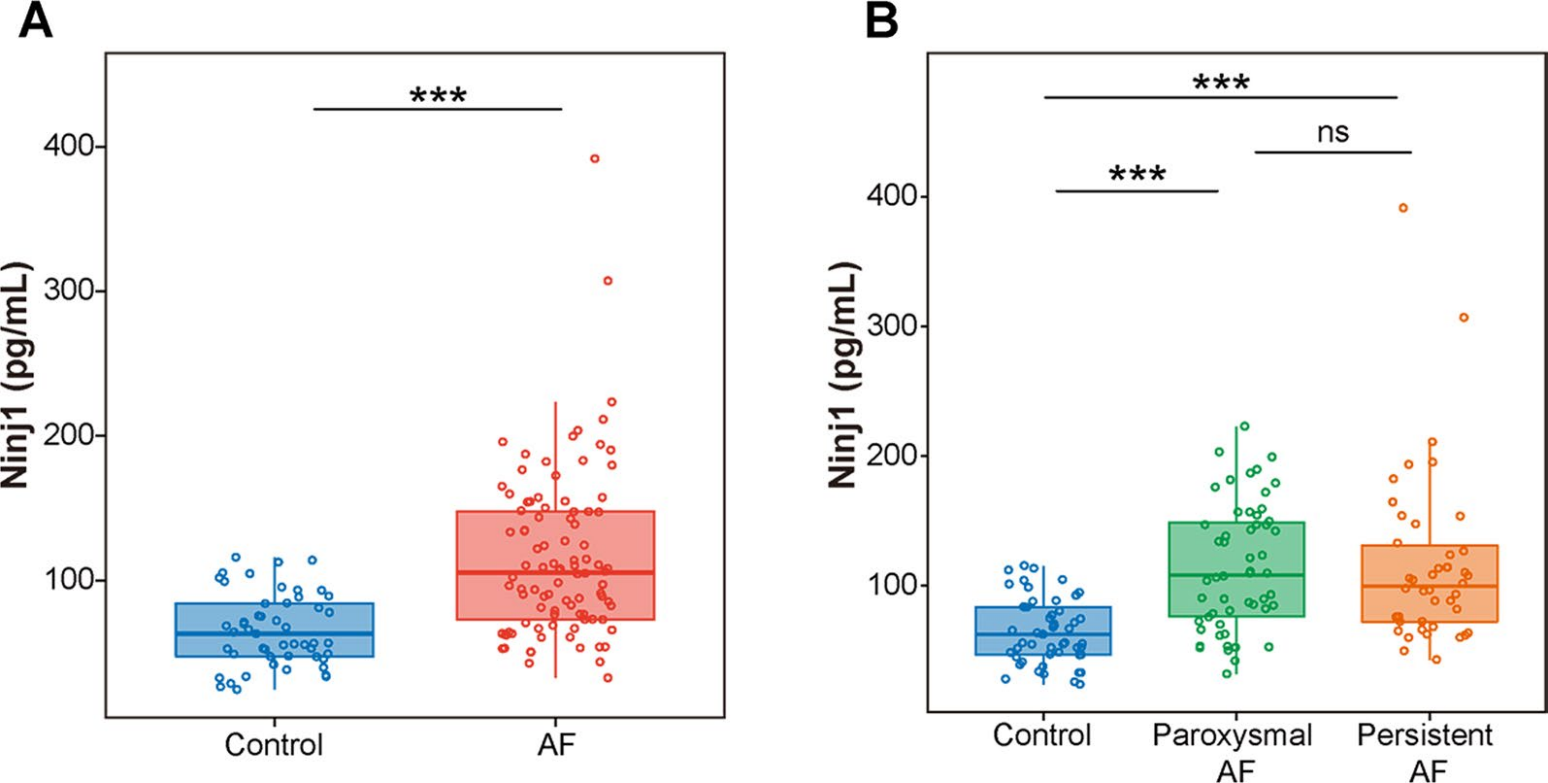
Ninj1 plasma levels in participants with or without AF. **A** Higher plasma Ninj1 levels in patients with AF than in controls. ***P < 0.001, Student's *t*-test. **B** Plasma Ninj1 levels in participants with different types of AF and controls. ***P < 0.001; ns, no significance. Student's *t*-test

The comparison in plasma Ninj1 levels between AF patients and controls revealed that the plasma Ninj1 levels were significantly elevated in AF patients (Control vs. AF: 65.40 ± 24.99 vs. 115.59 ± 57.39 pg/mL, *P* < 0.001) (Fig. 1A). In subgroup analysis, plasma Ninj1 levels were similar in patients with paroxysmal and persistent AF (114.67 ± 48.49 vs. 116.77 ± 67.74 pg/mL, *P* = 0.860), which were distinctly higher than controls (Control vs. Paroxysmal AF, *P* < 0.001; Control vs. Persistent AF, *P* < 0.001) (Fig. 1B). The baseline characteristics of the subgroup are depicted in Additional file 1: Table S1. To explore the effect of rhythm status at the time of assessment, we compared plasma Ninj1 levels between AF patients with AF and sinus rhythm and found no statistical difference between two rhythm statuses (Sinus rhythm vs. AF: 109.64 ± 45.05 vs. 120.02 ± 65.14 pg/mL, *P* = 0.383).

### Association of plasma Ninj1 levels with AF

**Fig. 2:**
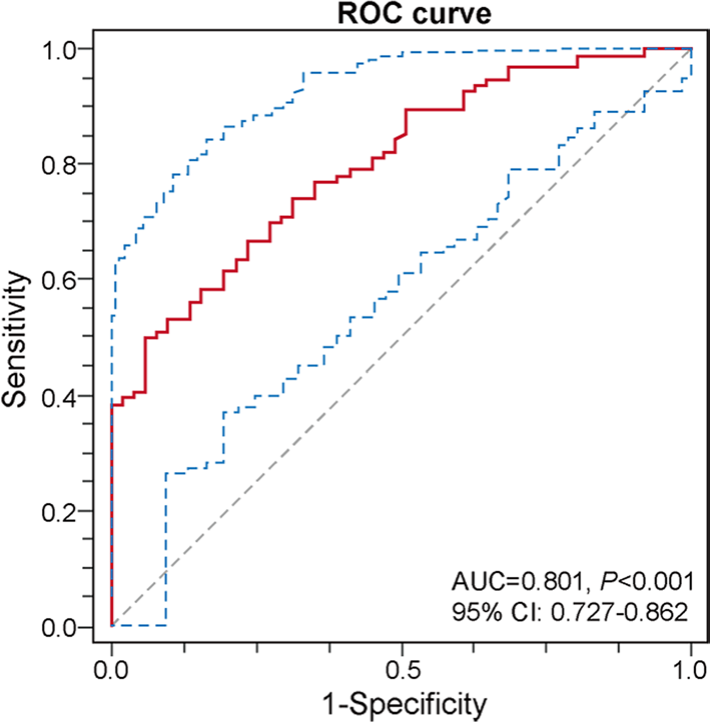
ROC for evaluating the association between plasma Ninj1 and AF

The results of the correlation analyses between plasma Ninj1 levels and AF-related clinical parameters based on Pearson’s and Spearman’s tests are represented in Table 2. Plasma Ninj1 was positively associated with age (R = 0.179, *P* = 0.030) and LAVI (R = 0.219, *P* = 0.019) and negatively related to LVEF (R = -0.284, *P* = 0.001). However, plasma Ninj1 exerted no significant correlation with AF history (R = 0.007, *P* = 0.946) and duration of persistent AF (R = 0.054, *P* = 0.735) in the AF group. Furthermore, multivariate logistic regression analysis was based on underlying AF risk factors, including gender, DM, age, HGB, HDL-C, LVEF, LAVI, and Ninj1 selected by univariate analysis (*P* < 0.100), and CHA2DS2-VASc score. As shown in Table 3, plasma Ninj1 levels and LAVI were significantly associated with AF. Notably, receiver operating characteristic (ROC) analysis indicated a higher predictive value of AF for plasma Nnij1 [area under the curve (AUC) = 0.801, 95% confidence interval (CI): 0.727–0.862; *P* < 0.001] (Fig. 2) with the optimal cut-off value of 105.13 pg/mL (sensitivity = 0.500, specificity = 0.941), than other clinical parameters related to AF progression [9–11] including neutrophil-to-lymphocyte ratio (NLR) (AUC = 0.627, 95% CI: 0.533–0.714) platelet-to-lymphocyte ratio (PLR) (AUC = 0.643, 95% CI: 0.550–0.729), high-sensitivity C-reactive protein (hs-CRP) (AUC = 0.550, 95% CI: 0.449–0.647) and LAVI (AUC = 0.758, 95% CI: 0.670–0.833) (Additional file 2: Fig. S1).

**Table 2:** Correlation between plasma Ninj1 and clinical variables

	R	*P* value
Age,years	0.179	0.030*
Male	− 0.002	0.978
BMI, kg/m^2^	− 0.153	0.100
Systolic blood pressure, mmHg	0.081	0.354
Diastolic blood pressure, mmHg	0.116	0.182
NLR	− 0.059	0.523
TC, mmol/L	0.052	0.549
TG, mmol/L	− 0.077	0.373
LDL-C, mmol/L	0.028	0.744
HDL-C, mmol/L	0.075	0.385
cTNI, ng/mL	− 0.025	0.789
ESR, mm/h	0.096	0.336
HbA1c, %	0.152	0.106
LVEF, %	− 0.284	0.001**
LAVI, ml/m^2^	0.219	0.019*

BMI, body mass index; cTNI, cardiac troponin I; ESR, erythrocyte sedimentation rate; HDL-C, high density lipoprotein cholesterol; HbA1c, hemoglobin A1c; LDL-C, low density lipoprotein cholesterol; LVEF, left ventricular ejection fraction; LAVI, left atrial volume index; NLR, neutrophil-to-lymphocyte ratio; TC, total cholesterol; TG, triglyceride. *, *P* < 0.05; **, *P* < 0.01

**Table 3:** Association between clinical characteristics and atrial fibrillation

	Univariate analysis		Multivariate analysis	
	OR (95% CI)	*P* value	OR (95% CI)	*P* value
Male	2.000 (1.004, 3.986)	0.049*		
DM	0.428 (0.171, 1.070)	0.070		
Age	1.049 (1.012, 1.088)	0.010*		
HGB	1.037 (1.006,1.069)	0.019*		
HDL-C	0.355 (0.109,1.160)	0.086		
LVEF	0.875 (0.806, 0.951)	0.002**		
CHA2DS2-VASc	1.157 (0.904, 1.481)	0.248		
LAVI	1.073 (1.035, 1.112)	< 0.001***	1.181 (1.049,1.330)	0.006**
Ninj1	1.037 (1.022,1.052)	< 0.001***	1.031 (1.008,1.056)	0.009**

DM, diabetes mellitus; HGB, hemoglobin; HDL-C, high density lipoprotein cholesterol; LVEF, left ventricular ejection fraction; LAVI, left atrial volume index

*P < 0.05; **P < 0.01; ***P < 0.001

### Correlation between plasma Ninj1 and thromboembolic risk in AF

**Fig. 3:**
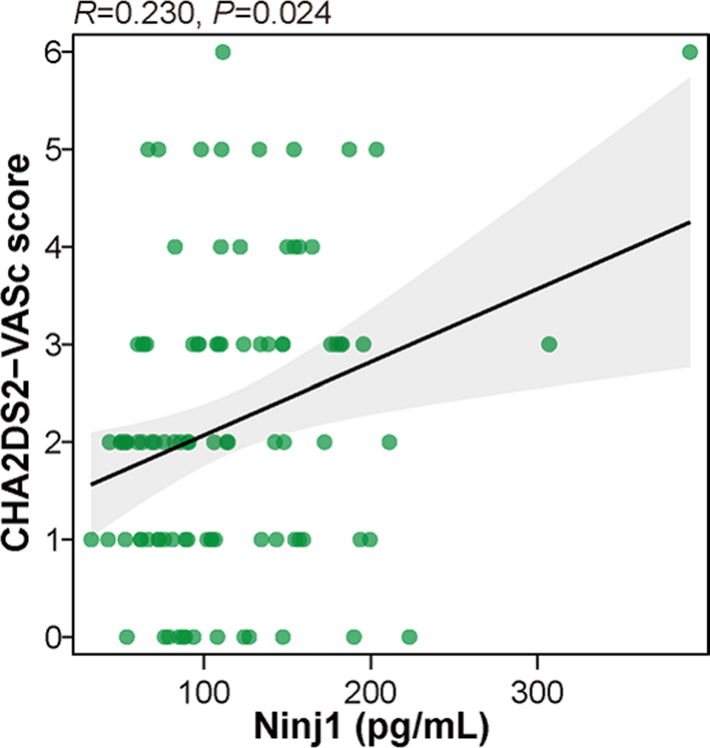
Correlation between plasma Ninj1 levels and CHA2DS2-VASc scores in patients with AF

AF is closely associated with increased thromboembolic and bleeding risks [12]. We further evaluated the correlation of plasma Ninj1 levels with CHA2DS2-VASc and HAS-BLED scores in patients with AF, which are recognized as risk assessment criteria for thromboembolism and bleeding, respectively. Interestingly, plasma Ninj1 was positively related to CHA2DS2-VASc score in AF patients with statistical significance (R = 0.230, *P* = 0.024) (Fig. 3), whereas plasma Ninj1 was non-significantly correlated with HAS-BLED score (R = 0.055, *P* = 0.597).

## Discussion

The present study showed elevated plasma Ninj1 levels in patients with AF for the first time. Plasma Ninj1 was positively correlated with LAVI and CHA2DS2-VASc score, which are crucial parameters of atrial remodeling and thromboembolism, respectively. As an independent risk factor, plasma Ninj1 exerts a prediction of AF.

Ninj1 is a double-transmembrane cell surface protein and contains two transmembrane regions with N- and C-termini outside the cytoplasm [13]. Ninj1 is wildly expressed in various tissues and cell types, including macrophages, leukocytes, and endothelial cells [14, 15]. The overexpression of Ninj1 promotes leukocyte infiltration and secretion of proinflammatory factors, including interleukin(IL)-6 [14]. A recent study demonstrated that Ninj1 plays a crucial role in inducing plasma membrane rupture during lytic cell death and releasing damage-associated molecular patterns (DAMPs) in mice macrophages. DAMP release is a key event in inflammation, which is bound with pyroptosis, apoptosis, and necrosis [13]. As an adhesion molecule, Ninj1 mediates cell–cell interaction through homotypic binding of an adhesive segment (26–37 amino acids) [16]. Furthermore, Ninj1 killing and adhesion rely on the structural integrity of the α-helix domain and the adhesive segment at the N-terminal regions outside the cytoplasm, respectively [13, 16]. The Ninj1-blocking peptide exerts anti-inflammatory and anti-apoptotic effects in septic and DM animal models [17, 18]. Notably, the N-terminal ectodomain of Ninj1 could be cleaved by MMP9, and the liberated soluble fragment has chemotactic activity [6]. The soluble Ninj1 suppresses cell adhesion [7]. Ninj1 dodecamer peptide containing N-terminal adhesion motif (Pro26–Asn37) exerts neuroprotection and anti-inflammation in the rat post-ischemic brain [19]. Furthermore, sNinj1-mimic peptides inhibit macrophage inflammatory response and monocyte recruitment irrespective of the presence of Ninj1 and are a secreted atheroprotective protein [8].

Inflammation and fibrosis are involved in the AF occurrence and development. Some studies reported that Ninj1 is upregulated in many inflammatory diseases, such as multiple sclerosis, rheumatoid arthritis, pulmonary fibrosis, and atherosclerosis [5, 8, 20, 21]. Similarly, we found that sNinj1 is elevated in AF, but no significant difference was observed in paroxysmal and persistent AF. Remarkably, sNinj1 in plasma was positively correlated to the thromboembolic risk of AF and LAVI but negatively related to LVEF. Also, LAVI is a superior parameter of left atrial size in predicting cardiovascular outcomes and AF recurrence [22, 23]. Left atrial enlargement is a critical substrate for AF [24]. These findings indicated that sNinj1 is associated with AF, thromboembolism, and atrial remodeling. In addition, plasma Ninj1 has a predictive utility and is an underlying biomarker for AF. Although the mechanism underlying the interaction between sNinj1 and AF is unknown, sNinj1 may be a promising therapeutic target for AF. However, further studies are required in the future.

Nevertheless, the present study has some limitations. First, this is a monocentric study, and the sample size is small, which might induce some selection bias. Second, since this is a cross-sectional study, the causative relation and specific mechanism between plasma Ninj1 and AF remain unclear.

## Conclusions

The current study revealed that plasma Ninj1 levels in AF patients are significantly increased. Plasma Ninj1 was positively correlated with left atrial enlargement and thromboembolic risk in AF patients.

## Supplementary Information

**Additional file 1: Table S1**. Baseline clinical characteristics of patients with atrial fibrillation.

**Additional file 2: Fig. S1**. ROC analysis on the correlation between clinical parameters and AF.

## Data Availability

The datasets generated and/or analysed during the current study are not publicly available due to the protection of patient privacy, but are available from the corresponding author on reasonable request.

## Abbreviations

AF: Atrial fibrillation
BMI: Body mass index
CAD: Coronary artery disease
DM: Diabetes mellitus
DAMP: Damage-associated molecular pattern
HGB: Hemoglobin
HTN: Hypertension
HDL-C: High density lipoprotein cholesterol
hs-CRP: High-sensitivity C-reactive protein
LAVI: Left atrial volume index
LVEF: Left ventricular ejection fraction
MMP9: Matrix metalloproteinase 9
Ninj1: Ninjurin-1
NLR: Neutrophil-to-lymphocyte ratio
PLR: Platelet-to-lymphocyte ratio
TC: Total cholesterol
