# Supplementary material for: Elevated plasma Ninjurin-1 levels in atrial fibrillation is associated with atrial remodeling and thromboembolic risk
Source: BMC Cardiovasc Disord. 2022 Apr 7;22:153. doi: 10.1186/s12872-022-02593-x (PMC8991886; doi:10.1186/s12872-022-02593-x)
Supplement: Supplementary file 1 — Additional file 1: Table S1. Baseline clinical characteristics of patients with atrial fibrillation. [file 12872_2022_2593_MOESM1_ESM.docx]

**Table S1**

Baseline clinical characteristics of patients with atrial fibrillation.

|  | **Paroxysmal AF** | **Persistent AF** | ***P* value** |
| --- | --- | --- | --- |
| Number | 54 | 42 | - |
| Male (%) | 28 (51.85%) | 28 (66.67%) | 0.210 |
| HTN (%) | 29 (53.70%) | 29 (69.05%) | 0.145 |
| DM (%) | 12 (22.22%) | 14 (33.33%) | 0.253 |
| CAD (%) | 5 (9.26%) | 5 (11.90%) | 0.744 |
| Smoking (%) | 8 (14.81%) | 9 (21.43%) | 0.430 |
| Drinking (%) | 7 (12.96%) | 9 (21.43%) | 0.285 |
| Age, years | 65.46±9.14 | 65.05±8.88 | 0.824 |
| BMI, kg/m^2^ | 25.04±3.14 | 26.49±3.29 | 0.047* |
| AF history, years | 2.00 (0.46, 5.00) | 3.00 (0.43, 7.00) | 0.566 |
| WBC, ×10^9^/L | 5.95±1.45 | 6.17±1.42 | 0.457 |
| HGB, g/L | 133.67±14.69 | 145.33±16.08 | <0.001*** |
| PLT, ×10^9^/L | 204.69±57.58 | 198.36±63.85 | 0.612 |
| TC, mmol/L | 3.97±0.95 | 4.15±0.80 | 0.322 |
| TG, mmol/L | 1.07 (0.77, 1.42) | 1.20 (0.93, 1.44) | 0.973 |
| LDL-C, mmol/L | 2.51±0.91 | 2.77±0.80 | 0.155 |
| HDL-C, mmol/L | 1.10±0.35 | 1.08±0.27 | 0.722 |
| AST, U/L | 21.00 (15.00, 23.00) | 17.00 (15.00, 20.00) | 0.190 |
| ALT, U/L | 21.00 (12.00, 26.00) | 16.00 (14.00, 20.00) | 0.753 |
| sCr, μmol/L | 69.17±14.97 | 73.28±14.92 | 0.184 |
| cTNI, ng/mL | 0.00 (0.00, 0.01) | 0.00 (0.00, 0.01) | 0.810 |
| HbA1c (%) | 5.90 (5.65, 6.35) | 6.10 (5.80, 6.90) | 0.071 |
| LVEF (%) | 64.00 (62.00, 69.00) | 64.00 (59.00, 68.00) | 0.051 |
| LAVI, ml/m^2^ | 20.83 (16.57, 27.20) | 32.08 (26.94, 35.22) | <0.001*** |
| ESR, mm/h | 4.00 (2.00, 11.00) | 5.00 (2.00, 10.00) | 0.487 |

Data are presented as mean ± SD, median (quartile) or number (%). ALT, alanine aminotransfease; AST, aspartate aminotransferase; BMI, body mass index; cTNI, cardiac troponin I; CAD, coronary artery disease; DM, diabetes mellitus; ESR, erythrocyte sedimentation rate; HTN, hypertension; HDL-C, high density lipoprotein cholesterol; HGB, hemoglobin; HbA1c, hemoglobin A1c; LDL-C, low density lipoprotein cholesterol; LVEF, left ventricular ejection fraction; LAVI, left atrial volume index; PLT, platelet; sCr, serum creatinine; TC, total cholesterol; TG, triglyceride; WBC, white blood cell. *, *P*<0.05; **, *P*<0.01; ***, *P*<0.001.
