# Supplementary figures and images for: Elevated plasma Ninjurin-1 levels in atrial fibrillation is associated with atrial remodeling and thromboembolic risk
Source: BMC Cardiovasc Disord. 2022 Apr 7;22:153. doi: 10.1186/s12872-022-02593-x (PMC8991886; doi:10.1186/s12872-022-02593-x)

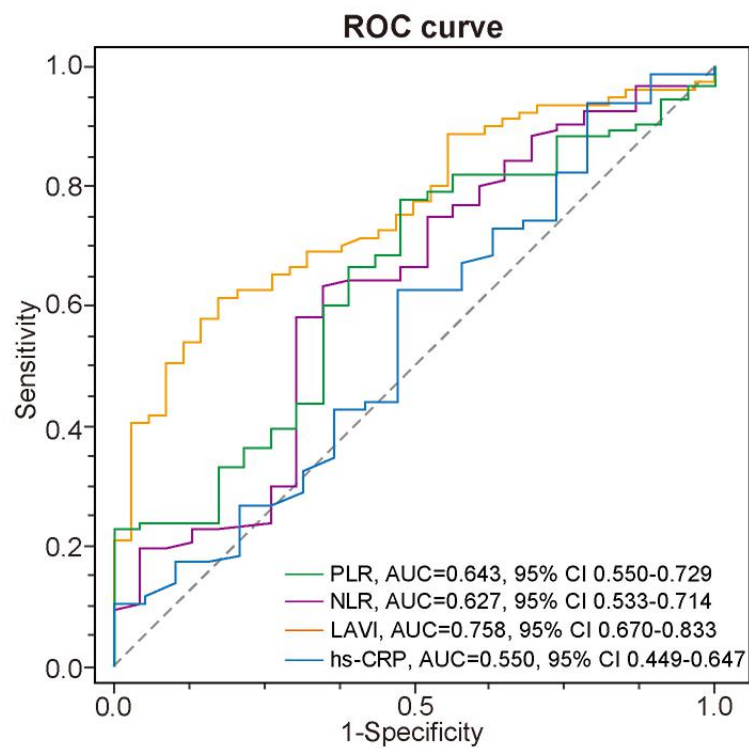

**Fig. S1** ROC analysis on the correlation between clinical parameters and AF.

Supplement: Supplementary file 2 — Additional file 2: Fig. S1. ROC analysis on the correlation between clinical parameters and AF. [file 12872_2022_2593_MOESM2_ESM.pdf]
